# CircSOX9 acts as a molecular sponge of miR-485-3p to promote the progression of nasopharyngeal carcinoma

**DOI:** 10.18632/aging.204127

**Published:** 2022-06-14

**Authors:** Yanbo Sun, Yun Liu, Zhihui Du, Liangqiang Zhou, Qingguo Chen, Hanqi Chu

**Affiliations:** 1 Department of Otolaryngology-Head and Neck Surgery, Tongji Hospital, Tongji Medical College, Huazhong University of Science and Technology, Wuhan 430000, People’s Republic of China

**Keywords:** nasopharyngeal carcinoma, circular RNA, ceRNA, circSOX9, miR-485-3p, SOX9

## Abstract

Circular RNA (circRNA) plays a vital role in the occurrence and development of nasopharyngeal carcinoma (NPC). However, the role of certain specific circRNAs in NPC are still unknown. In this study, collect tumor samples and adjacent normal tissues from clinical NPC patients and detect the expression of circSOX9 by qRT-PCR. Use nucleoplasmic separation analysis, RNase R digestion assay and FISH to detect the characteristics of circSOX9. After knocking down circSOX9, clone formation experiment and transwell assay were used to detect the proliferation and invasion ability of nasopharyngeal carcinoma cells HONE1 and CNE2, and western blot was used to further detect the level of epithelial-mesenchymal transition (EMT). Use the database to screen for possible downstream target genes and verify them with dual-luciferase experiments. Bioinformatics analysis showed that circSOX9 was significantly up-regulated in NPC, and its expression level was positively correlated with the malignant progression of cancer. Data from function gain or loss studies showed that decrease of circSOX9 inhibited the invasion and proliferation of HONE1 and CNE2 cell lines. Further analysis proved that miR-485-3p was the downstream target of circSOX9. The luciferase test showed that by acting as a molecular sponge of miR-485-3p, circSOX9 promotes the proliferation and invasion of NPC cells, while miR-485-3p can target the expression of SOX9. In conclusion, circSOX9 acts as an oncogene in the progression of NPC through miR-485-3p/SOX9, indicating that circSOX9 can be used as a potential therapeutic target and predictive marker for nasopharyngeal carcinoma.

## INTRODUCTION

Nasopharyngeal carcinoma (NPC) is a highly prevalent malignant tumor in China [1]. Nasopharyngeal carcinoma has the pathological characteristics of rapid deterioration and poor prognosis, which often causes most patients to have developed to the middle and advanced stages at the time of diagnosis [2, 3]. Until recently, the underlying pathways and underlying mechanisms of NPC were unclear. The ability to characterize the cancer genome now provides insight into the origin and molecular basis of this disease.

CircRNA is a type of non-coding RNA that has been identified as an important gene expression regulator and is involved in cancer progression [4]. CircRNA mainly regulates cell state in three ways: encoding small peptides, ceRNA or directly binding to RBP protein [5, 6]. During the occurrence and development of tumors, many circRNAs participate in a series of biological processes related to invasion, metastasis, and proliferation [7, 8]. However, the role of circRNA in NPC is rarely reported.

Here, we examined the differentially expressed circRNA that was significantly up-regulated in NPC tissues and found that knockdown of circSOX9 significantly reduced its malignancy. Bioinformatics analysis showed that circSOX9 was significantly up-regulated in NPC, and its expression level was positively correlated with the malignant progression of cancer. Data from function gain or loss studies showed that decrease of circSOX9 inhibited the invasion and proliferation of HONE1 and CNE2 cell lines. Further analysis proved that miR-485-3p was the downstream target of circSOX9. The luciferase test showed that by acting as a molecular sponge of miR-485-3p, circSOX9 promotes the proliferation and invasion of NPC cells, while miR-485-3p can target the expression of SOX9. We studied the possible targeting of miRNA and noticed that circSOX9 targets miR-485-3p/SOX9 like ceRNA and thus enhances the progression of nasopharyngeal carcinoma.

## RESULTS

### CircSOX9 is increased in NPC cells and tissues

**Figure 1:**
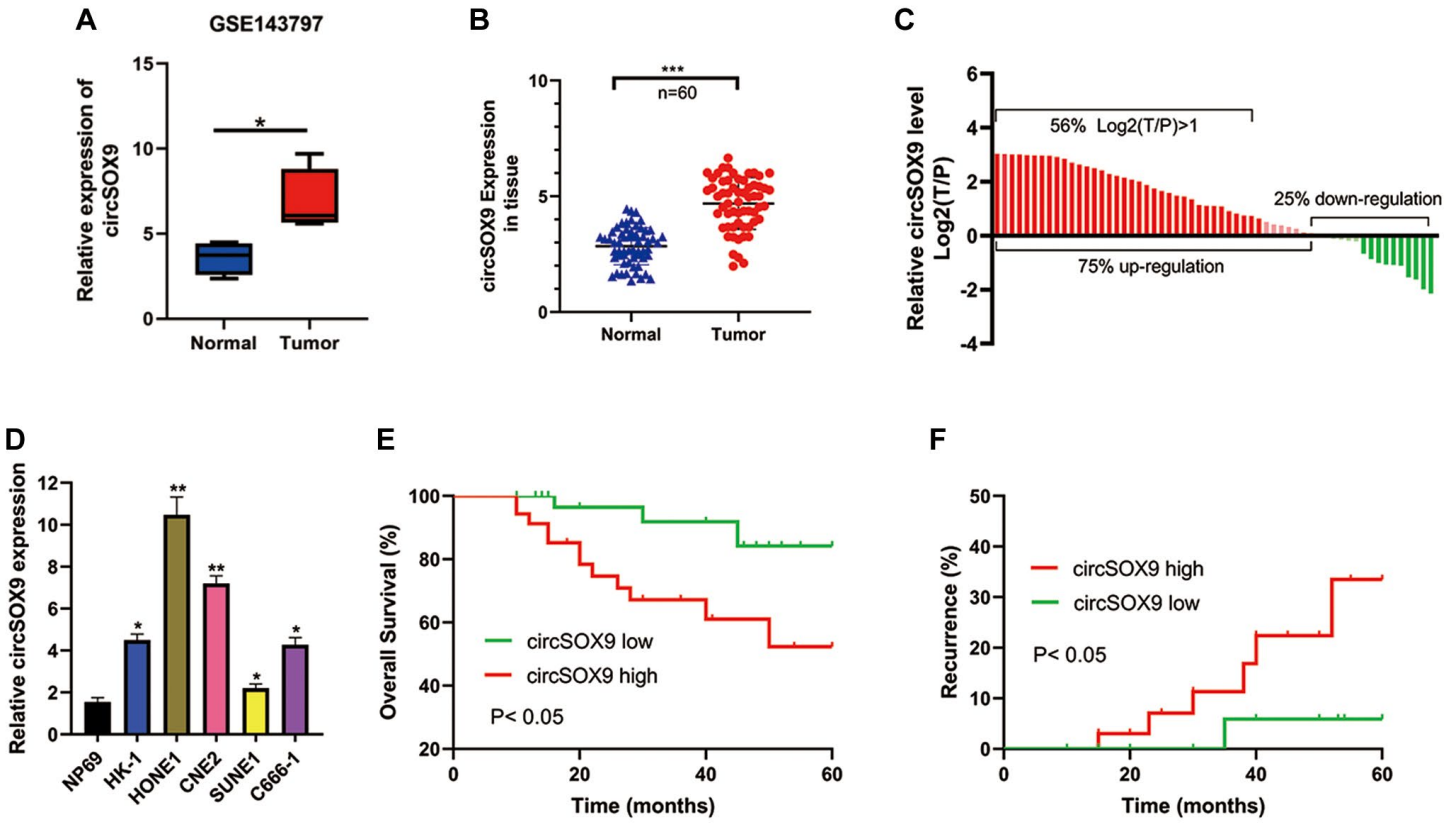
**CircSOX9 is increased in NPC cells and tissues.** (**A**) Bioinformatic analysis the circSOX9 expression in the GEO database (GSE143797). (**B**) The tumor tissues and para-tumor tissues of 60 patients were collected, and the expression difference of circSOX9 was detected by qRT-PCR. (**C**) Count the expression of NPC and para-tumor in each patient, T/P: tumor vs para-tumor. (**D**) qRT-PCR was used to detect the expression of circSOX9 in NPC cell lines NP69, HK-1, HONE1, CNE2, SUNE1, and C666-1. (**E**) The relationship between the expression of circSOX9 in tumor tissues and overall survival. (**F**) The relationship between the expression of circSOX9 in tumor tissues and its recurrence.^*^P < 0.05, ^**^P < 0.01, ^***^P < 0.001.

First, we analysis the different expressed circRNAs of NPC tissues in the GEO database (GSE143797) and found circSOX9 was significantly overexpressed in the NPC tissues (Figure 1A). Then, we detected the expression of circSOX9 in NPC tumor tissues and para-tumor tissues in 60 collected patients (Figure 1B). Table 1 shows the relationship between circSOX9 expression and clinical parameters. Divide the expression of circSOX9 in tumor tissues by the expression of circSOX9 in para-tumor tissues, and take the logarithm of 2. The results show that the expression of circSOX9 in tumor tissues of most patients with nasopharyngeal carcinoma is significantly higher than that in para-tumor tissues (Figure 1C). The expression levels of circSOX9 were then further investigated in NPC cell lines (Figure 1D). The HONE1 and CNE2 cell lines with the highest expression levels of circSOX9 were used in subsequent experiments. Statistics on the patient’s tumor recurrence and prognosis found that the high expression of circSOX9 is associated with a poor prognosis and a higher recurrence rate (Figure 1E, 1F, Table 1). The above indicates that circSOX9 is upregulated in NPC, which may be related to tumorigenesis and its progression.

**Table 1:** The correlation of clinical features and circSOX9 expression in NPC patients.

**Characteristics**	**Number of cases**	**circSOX9 expression**		***P* value**
		**Low (*n* = 30)**	**High (*n* = 30)**	
Gender				0.2839
Male	38	17	21	
Female	22	13	9	
Age (years)				0.4321
≤45	25	14	11	
>45	35	16	19	
Tumor size (cm)				0.2918
≤1	36	20	16	
>1	24	10	14	
Tumor stage				0.2598
T1-T2	18	11	7	
T3-T4	42	19	23	
Lymphatic metastasis				0.0384
Negative	28	18	10	
Positive	32	12	20	
Distant metastasis				0.0099
M0	43	26	17	
M1	17	4	13	
Clinical stage				0.0092
I–II	34	22	12	
III–IV	26	8	18	

### The characteristics of the circSOX9

**Figure 2:**
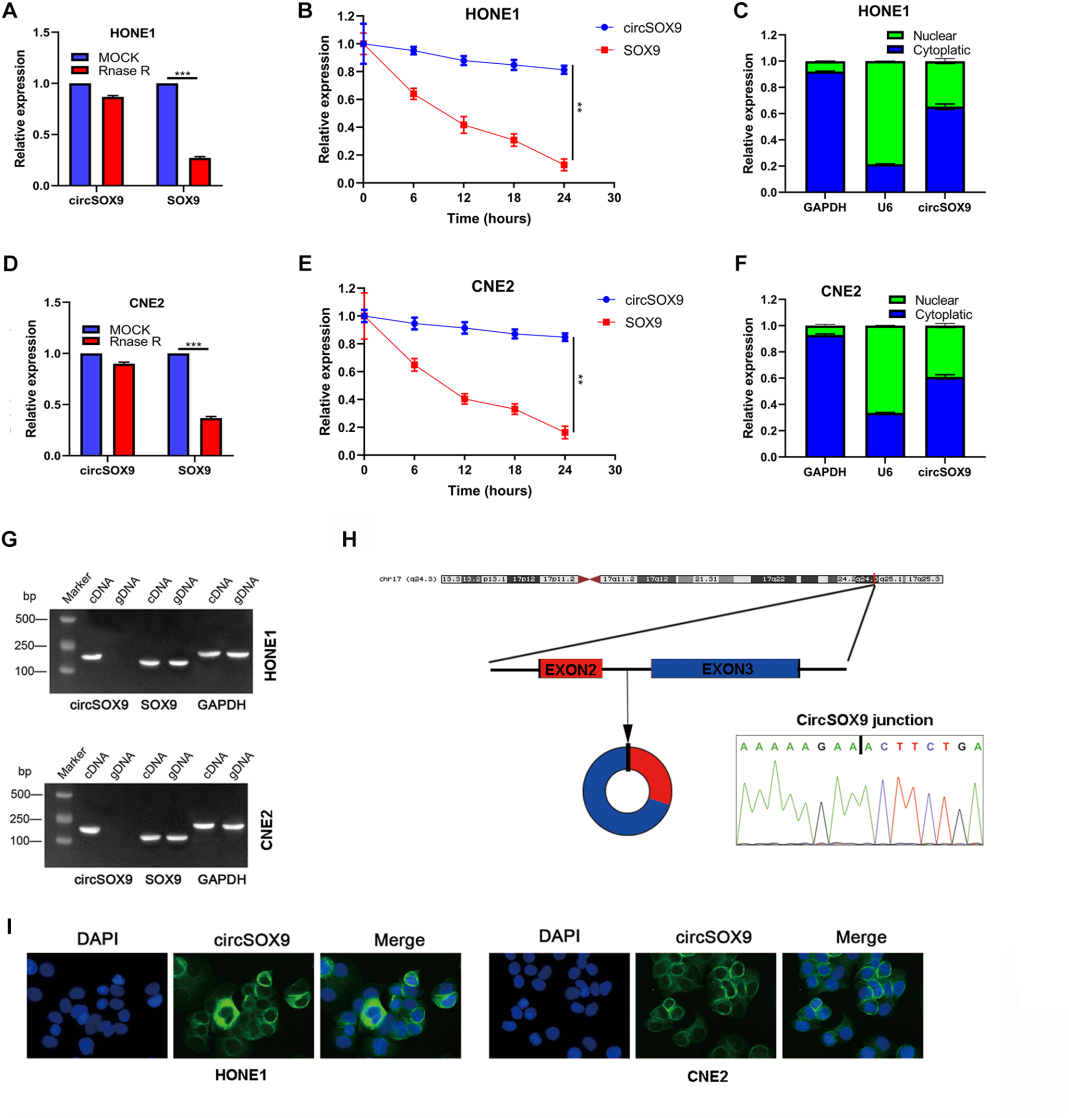
**Molecular characteristics and expression localization of circSOX9.** (**A**) RNase R digestion assay was used to detect the stability of circSOX9 in HONE1 cells. (**B**) The qRT-PCR analysis of circSOX9 and linear SOX9 was performed by RNase R digestion assay in HONE1 cells. (**C**) The location of circSOX9 was detected using nucleoplasmic separation analysis in HONE1 cells. (**D**) The stability of circSOX9 was evaluated by RNase R digestion assay in CNE2 cells. (**E**) qRT-PCR analysis for circSOX9 was and linear SOX9 by RNase R digestion assay in CNE2 cells. (**F**) The location of circSOX9 was detected using nucleoplasmic separation analysis in CNE2 cells. (**G**) qRT-PCR analysis for circSOX9 and linear Sox9 in cDNA and gDNA in HONE1 and CNE2 cells. (**H**) Sanger sequence analysis of the back splice sequence of circSOX9. (**I**) RNA FISH analysis showed that circSOX9 was mainly distributed in the cytoplasm, scale bar = 50 μm. ^**^P < 0.01, ^***^P < 0.001.

Before delving into the function of circSOX9 in NPC, it is necessary to investigate the characteristics of circSOX9. In the HONE1 cell line, circSOX9 is resistant to RNase R, while mSOX9 is significantly inhibited after RNase R treatment (Figure 2A, 2B). The analysis of cytoplasm and nuclear separation showed that most of circSOX9 were distributed in the cytoplasm (Figure 2C). We further repeated the above experiment in CNE2 (Figure 2D–2F). Figure 2G shows that circSOX9 was amplified by divergent primers, only from cDNA and not from gDNA.

Sanger sequence analysis showed the back splice sequence of circSOX9 (Figure 2H). RNA FISH analysis showed that circSOX9 is mainly distributed in the cytoplasm (Figure 2I). The above data shows that the circSOX9 sample we studied is round and mainly located in the cytoplasm.

### CircSOX9 promotes the proliferation and migration of HONE1 and CNE2 cells

**Figure 3:**
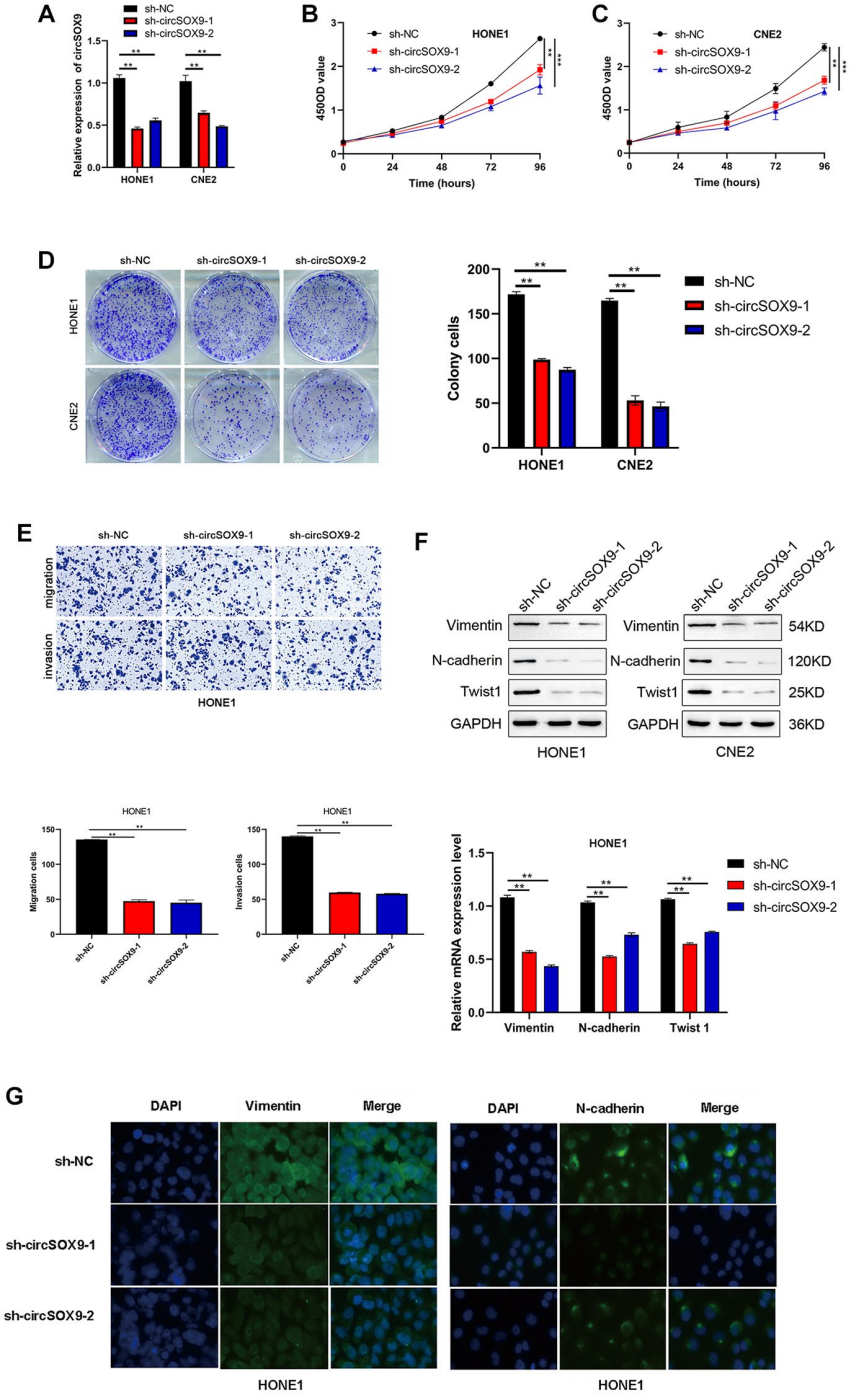
**CircSOX9 promotes the proliferation and migration of HONE1 and CNE2 cells.** (**A**) qRT-PCR was used to measure the effect of circSOX9 knockdown in HONE1 or CNE2 cells. (**B**, **C**) CCK8 detects cell proliferation ability of HONE1 and CNE2 after knocking down circSOX9. (**D**) Clone formation experiment detects the proliferation ability of HONE1 and CNE2 after knocking down circSOX9. (**E**) Transwell assay detects the invasion and migration ability of HONE1 after knocking down circSOX9. (**F**) After knocking down circSOX9 in HONE1 or CNE2 cells, qRT-PCR and western blot assays were used to detect the expression of EMT-related markers Vimentin, N-cadherin, and Twist1. (**G**) Immunofluorescence analysis of Vimentin and E-cadherin expression in NPC cells, scale bar = 50 μm. ^**^P < 0.01, ^***^P < 0.001.

**Figure 4:**
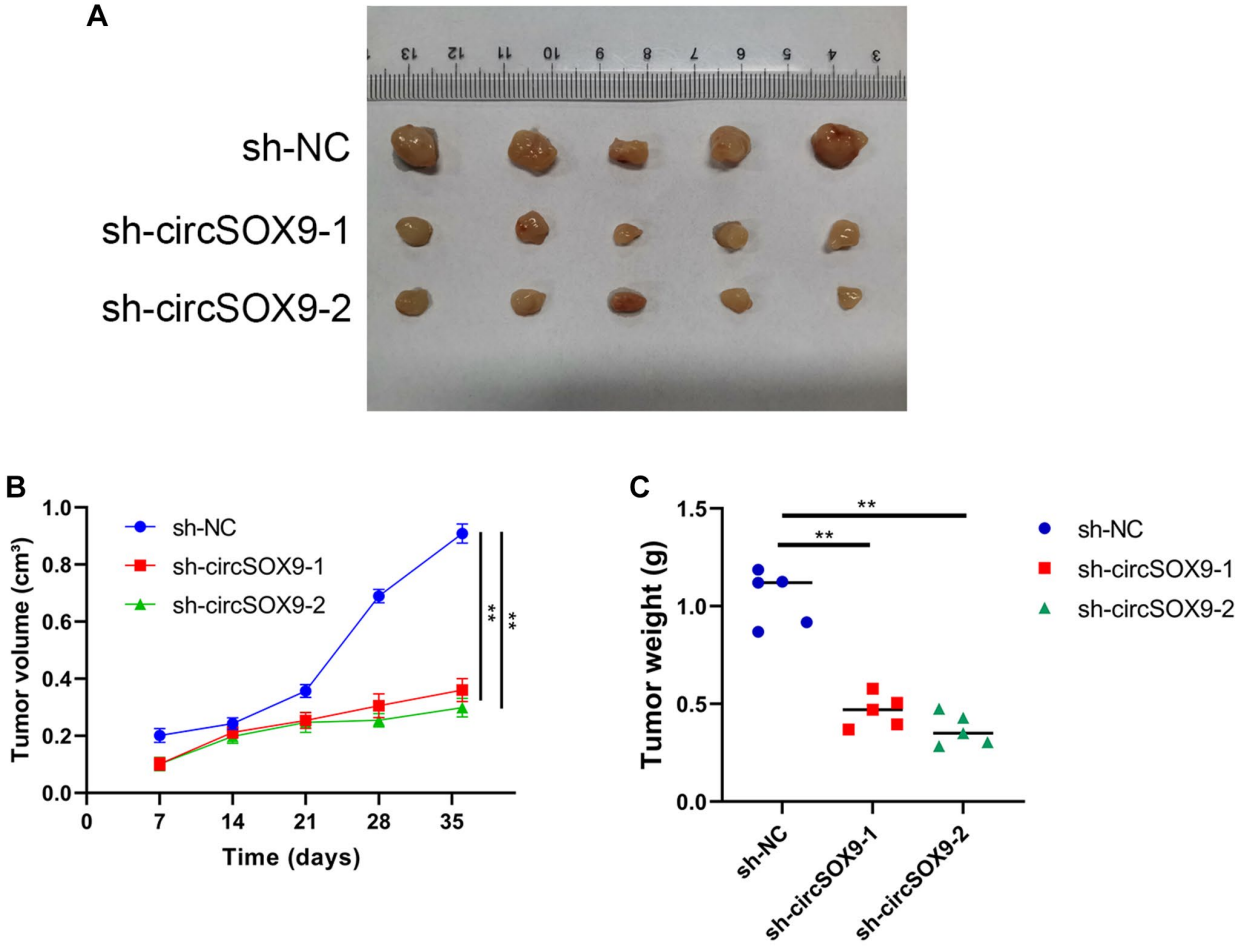
**CircSOX9 promotes the proliferation of NPC cell *in vivo*.** (**A**) Subcutaneous tumorigenesis experiments in nude mice were used to analyze the effect of circSOX9 on the proliferation ability of HONE1 cell. (**B**) Volume Growth Curve Analysis of Subcutaneous Tumors. (**C**) Weight analysis of subcutaneous tumors.^*^P < 0.05.

To investigate the functional role of circSOX9 in NPC cells, circSOX9 shRNA was constructed and transfected the plasmid into HONE1 and CNE2 cells. Use qRT-PCR to verify transfection efficiency (Figure 3A). Then the CCK8 assay was used to determine the proliferation ability after knocking down circSOX9 in HONE1 and CNE2 cells. Figures 3B, 3C show that knocking down circSOX9 significantly inhibits cell proliferation. Similar results were shown in the clone formation experiment, indicating that knocking down circSOX9 significantly inhibited the proliferation of HONE1 and CNE2 cells (Figure 3D). Figure 3E shows the invasion and migration ability of HONE1 cells measured by transwell assay. The results show that knocking down circSOX9 significantly inhibits the invasion and migration ability of HONE1 cells. Western blot analysis of markers related to epithelial-mesenchymal transition (EMT) showed that knocking down circSOX9 significantly inhibited the expression of Vimentin, N-cadherin, Twist1 (Figure 3F). Immunofluorescence analysis indicated that knocking down circSOX9 significantly inhibited the expression of Vimentin, N-cadherin in NPC cell (Figure 3G). *In vivo* experiment, we found that the growth ability of HONE cell was significantly inhibited in circSOX9 knockdown group (Figure 4A), and the volume and weigh also decreased compared to the control group (Figure 4B, 4C). The above data shows that circSOX9 can promote the proliferation and migration of NPC cells.

### Screening the potential target genes of circSOX9 in NPC cells

**Figure 5:**
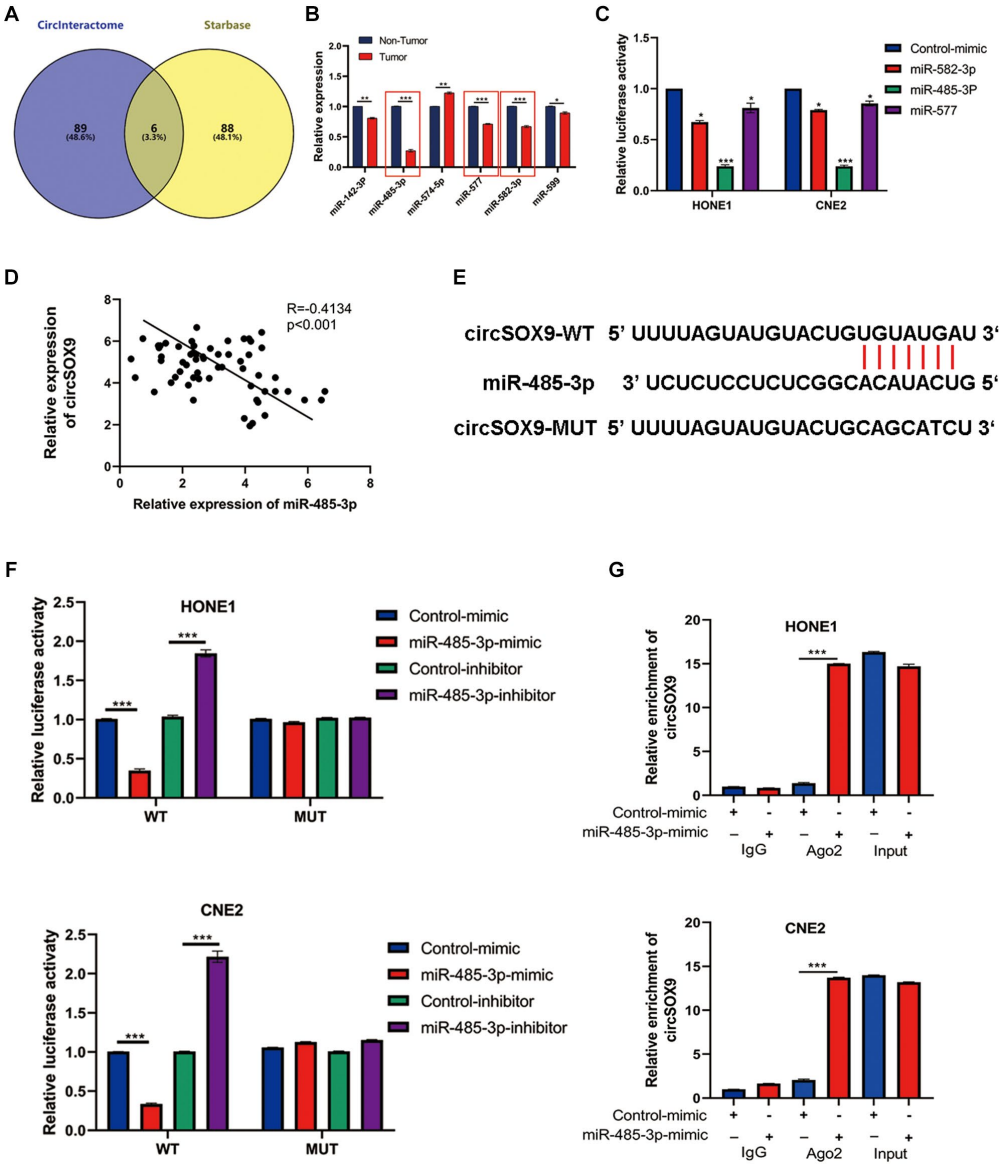
**MiR-485-3p was the sponge target of circSOX9 in NPC cells.** (**A**) Use predictive software (CircInteractome, Starbase) for bioinformatics analysis of potential target genes. (**B**) qRT-PCR analysis of target miRNAs in NPC tumors and para-tumor tissues. (**C**) Dual-luciferase reporter gene assay detects the interaction of circSOX9 and miR-485-3p, miR-577, and miR-582-3p. (**D**) qRT-PCR analysis of the expression correlation of circSOX9 and miR-485-3p in NPC. (**E**) The binding site between circSOX9-wt and miR-485-3p, and the mutant sequence (circSOX9-mut) that cannot bind to miR-485-3p was designed. (**F**) The dual-luciferase reporter gene assay proved the direct binding between circSOX9 and miR-485-3p in HONE1 and CNE2 cells. (**G**) RIP assay verified the combination of circsox9 and miR-485-3p with Ago2).^*^P < 0.05, ^**^P < 0.01, ^***^P < 0.001.

Predictive software (CircInteractome, Starbase) was used to screen out potential circSOX9 target genes. Six miRNAs were selected as putative target genes for follow-up studies (Figure 5A). Among the 6 miRNAs, we found that miR-485-3p, miR-577, and miR-582-3p were significantly downregulated in NPC tumor tissues (Figure 5B). The data from the luciferase assay suggested that the miR-485-3p could inhibit the luciferase activity of circSOX9 3′UTR (Figure 5C). Subsequently, qRT-PCR was used to detect the expression of miR-485-3p and circSOX9 in NPC tissues, then Person analysis of the expression levels indicated that circSOX9 and miR-485-3p were negatively correlated in NPC tissues (Figure 5D). Bioinformatic analysis indicated that miR-485-3p was downregulated and positive correlation with NPC prognosis based on GEO database (Supplementary Figure 1). Data from luciferase assay showed that miR-485-3p could combine with circSOX9 both in HONE1 and CNE2 cells (Figure 5E, 5F). The RIP test shows that Ago2 can bind to circSOX9 and miR-485-3p, indicating that these two can act on Ago2 (Figure 5G). Together, these results indicate that circSOX9 perhaps sponge miR-485-3p both in HONE1 and CNE2 cells.

### MiR-485-3p targets the SOX9 expression

**Figure 6:**
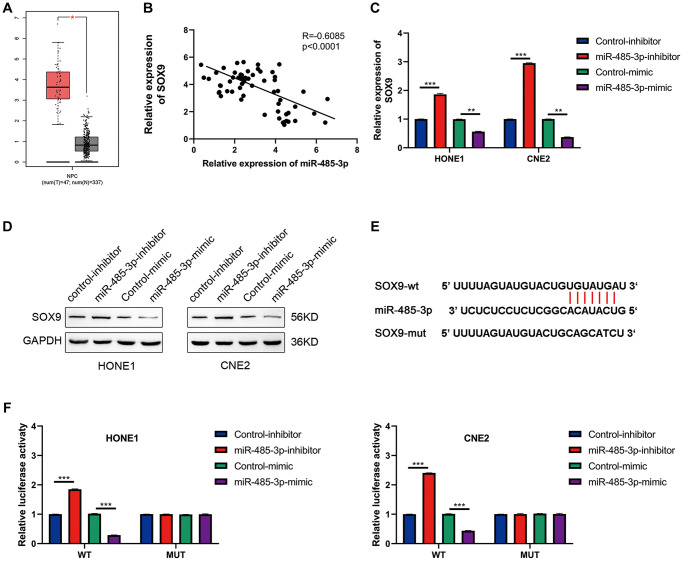
**SOX9 is the downstream target gene of miR-485-3p.** (**A**) The expression level of SOX9 in NPC and para-tumor tissues was analyzed based on the TCGA database. (**B**) The person analysis shows the miR-485-3p and circSOX9 expression correlation in NPC tissues. (**C**, **D**) qRT-PCR and Western blot analysis of SOX9 expression in HONE1 and CNE2 cells after miR-485-3p inhibition or overexpression. (**E**) The predicted binding site of miR-485-3p and SOX9. (**F**) Dual-luciferase reporter gene assay was used to prove the binding of SOX9 and miR-485-3p. ^*^P < 0.05, ^**^P < 0.01, ^***^P < 0.001.

After analyzing NPC using the TCGA database, we found that SOX9 was up-regulated in NPC tissues (Figure 6A). Further analysis showed that SOX9 and miR-485-3p were negatively correlated in NPC tissues (Figure 6B). Data from western blot and qRT-PCR assay showed that miR-485-3p may inhibit SOX9 expression in HONE1 and CNE2 cells (Figure 6C, 6D). Then we found the binding site of miR-485-3p and SOX9 through bioinformatics analysis and constructed a luciferase mutant plasmid based on the binding site (Figure 6E). Data from luciferase assay showed that miR-485-3p could bind with SOX9 3′UTR (Figure 6F). Together, these data indicated that SOX9 was the target of miR-485-3p in NPC cells.

### CircSOX9 promotes the proliferation and invasion of nasopharyngeal carcinoma by regulating miR-485-3p/SOX9 signal axis

**Figure 7:**
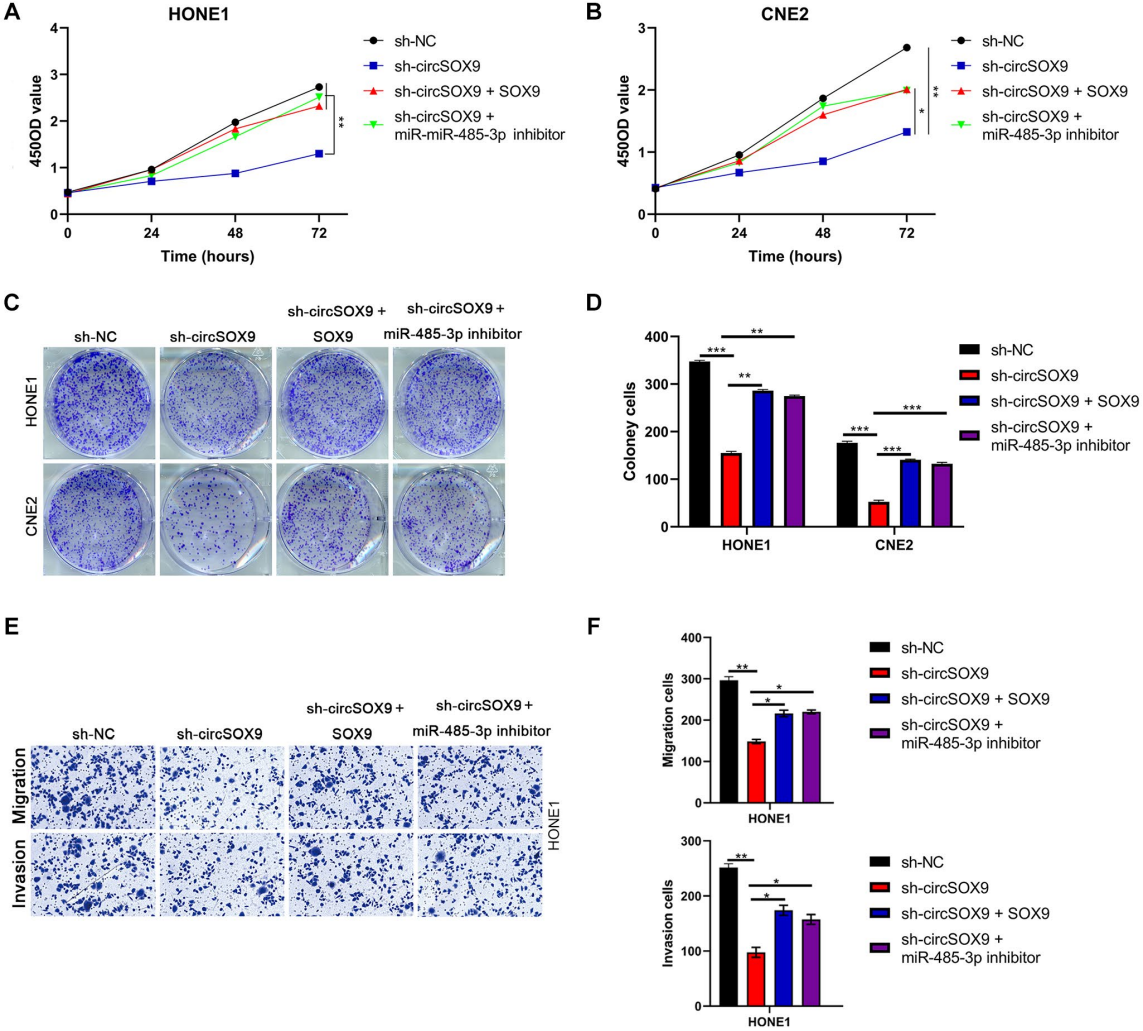
**CircSOX9 promotes the proliferation and invasion of nasopharyngeal carcinoma by regulating miR-485-3p/SOX9 signal axis.** (**A**, **B**) After HONE1 or CNE2 was transfected with the corresponding plasmid, cell proliferation of each group was tested by CCK8. (**C**, **D**) After HONE1 or CNE2 was transfected with the corresponding plasmid, cell proliferation of each group was tested by plate formation assays. (**E**, **F**) Transwell assay was used to detect cell migration and invasion in each group.^*^P < 0.05, ^**^P < 0.01, ^***^P < 0.001.

To further verify the existence of circSOX9/miR-485-3p/SOX9 axis in NPC cells. HONE1 or CNE2 cells were then transfected with sh-circSOX9 and miR-485-3p inhibitor or SOX9 overexpression plasmid. Data from plate colony and CCK8 assay showed that sh-circSOX9 can inhibit the proliferation of NPC cells, while miR-485-3p inhibitor and SOX9 overexpression plasmid can partially reverse the effect of sh-circSOX9 (Figure 7A–7D). Data from the transwell assay showed that invasion and migration of HONE1 cells were down-regulated after knocking down circSOX9 and rescued by transfection with miR-485-3p inhibitor or SOX overexpression plasmid (Figure 7E, 7F). Meanwhile, in cells with knockdown of miR-485-3p, the up-regulation of SOX9 by circSOX9 was suppressed (Supplementary Figure 2).

## DISCUSSION

According to reports, many genes produce individual circRNAs in different cells, but their biological functions are unclear. With the development of high-throughput RNA sequencing and bioinformatics analysis, the distribution map of human circRNAs in different cells and tissues has been published. Studies have shown that abnormally expressed circRNAs is related to clinical characteristics and may become a biomarker of tumor occurrence and development. In this study, we found that the circular RNA from the SOX9 exon (named circSOX9) was significantly up-regulated in NPC tissues and the high expression of NPC was positively correlated with the progress of NPC.

Our study proved the relationship between miR-485-3p and circSOX9. Both circRNA and miRNA are important components of non-coding RNA [9, 10]. In tumor research, circRNAs use their own binding sites as sponges to adsorb miRNAs, and regulate miRNAs through competitive endogenous RNAs (ceRNAs), thereby regulating the expression of downstream target genes [11, 12]. Our research shows that miR-485-3p targeted by circSOX9 is closely related to the proliferation and invasion ability of NPC cells HONE1 and CNE2. It has been reported that miR-485-3p is located in the 14q32.31 chromosome region [13]. A large number of studies have frequently observed cancer mutations in this region, which suggests that miR-485-3p may have tumor suppressor potential [14, 15]. Our research shows that the expression of miR-485-3p in NPC is down-regulated, and it exerts a tumor suppressor effect by targeting SOX9.

SOX9 is an important transcription factor that regulates the DNA binding and transactivation domains of the high-mobility group box [16]. However, the work of SOX9 in NPC has not been reported. Studies have pointed out that SOX9 regulates some of its target genes through the abundant transcription enhancer clusters in histone H3 acetylated by lysine 27 (H3K27ac) [17, 18]. SOX9 participates in the progression of many cancers through this model. SOX9 has previously been shown to be highly expressed in aggressive cancers and has also been identified as a negative prognostic factor for lung cancer [19–21]. However, the biological function of SOX9 in NPC remains unclear. Here we identified the miR-485-3p/SOX9 axis as a signaling pathway that promotes tumor progression in response to circSOX9. The data shows that SOX9 can promote the progress of NPC, and its expression is regulated by miR-485-3p.

## MATERIALS AND METHODS

### Tissue specimens

We collected tumor tissues and adjacent non-tumor (para-tumor) tissues of patients (*n* = 60) who underwent nasopharynx tumor biopsy and pathological diagnosis confirmed that it was nasopharyngeal carcinoma from March 2016 to March 2019. patient information is shown in Table 1. The study was approved by the ethics committee and all patients provided written informed consent.

### Cell culture and transfections

Human NPC cells NP69, HK-1, HONE1, CNE2, SUNE1, and C666-1 cells were cultured in DMEM containing 10% FBS and kept in 37°C and 5% CO_2_ incubator. Guangzhou Ribo Biotechnology provided circSOX9 shRNA, miR-485-3p mimic plasmids, and negative controls. Transfect siRNA, miRNA mimics, and miRNA inhibitors into HONE1 or CNE2 cells used in Lipofectamine 3000. Then the western blot assay was used to verify the plasmid efficiency.

### Western blot analysis

HONE1 or CNE2 cells in each group were washed 3 times with cold PBS, and then lysed for 30 minutes. The protein concentration was then measured using the BSA kit. The measured protein was then loaded and separated by electrophoresis in a 10% SDS-PAGE gel and transferred to the PVDF membrane. Incubate the PVDF membrane with the corresponding primary antibody overnight, wash the membrane three times with TBST, incubate the membrane with the corresponding secondary antibody at room temperature for 1 h, wash the membrane three times with TBST, and detect by chemiluminescence.

### Luciferase and dual-luciferase assay

The plasmid psiCHECK contains the cloned circSOX9 WT and its mutant sequence. HONE1 or CNE2 cells (4 × 10^4^ cells/well) were cultured overnight in a 6-well plate and transfected with psiCHECKCirc-SOX9 mutant, psiCHECK-circSOX9 WT and psiCHECK vector for Renilla luciferase expression. Cells were lysed and the luciferase activity was studied by the dual-luciferase gene detection system after 24 h.

### Transwell assay

Use 8 μm-well Transwell inserts to determine the invasiveness of the cells. Inoculate 100 μL of cells (2 × 10^5^) in serum-free medium into the upper chamber and 400 μL of medium containing 10% FBS in the lower chamber. After culturing in an incubator for 24 hours, the cells were fixed with methanol, stained with 0.1% crystal violet, and photographed and counted under an inverted microscope.

### Statistical analysis

One-way analysis of variance (ANOVA) was used to analyze differences between groups, and all data were expressed as mean ± SEM. ^*^p < 0.05 ^**^p < 0.01 ^***^p < 0.001 is considered statistically significant.

### Data availability

Data supporting the findings of this study are available from the corresponding author upon request.

## Supplementary Materials

Supplementary Figures
