# Supplementary material for: CircSOX9 acts as a molecular sponge of miR-485-3p to promote the progression of nasopharyngeal carcinoma
Source: Aging (Albany NY). 2022 Jun 14;14(11):4914–26. doi: 10.18632/aging.204127 (PMC9217694; doi:10.18632/aging.204127)
Supplement: Supplementary Figures [file aging-14-204127-s001.pdf]

SUPPLEMENTARY FIGURES

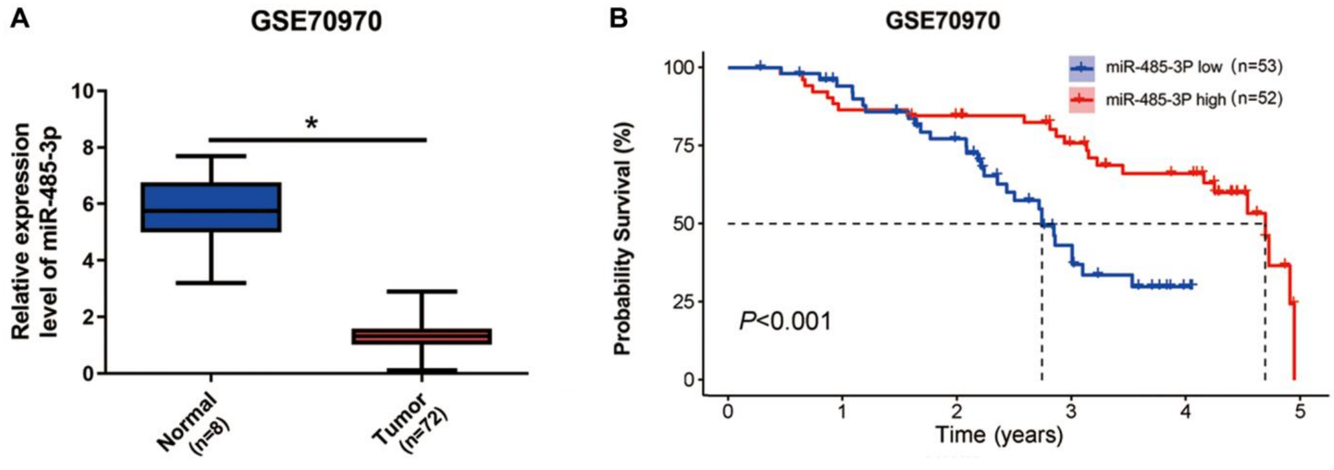

Supplementary Figure 1. (**A**) GEO database analysis of the expression of miR-485-3p in NPC tissues. (**B**) GEO database analysis of the correlation of expression of miR-485-3p and NPC prognosis. $^{*}P < 0.05$.

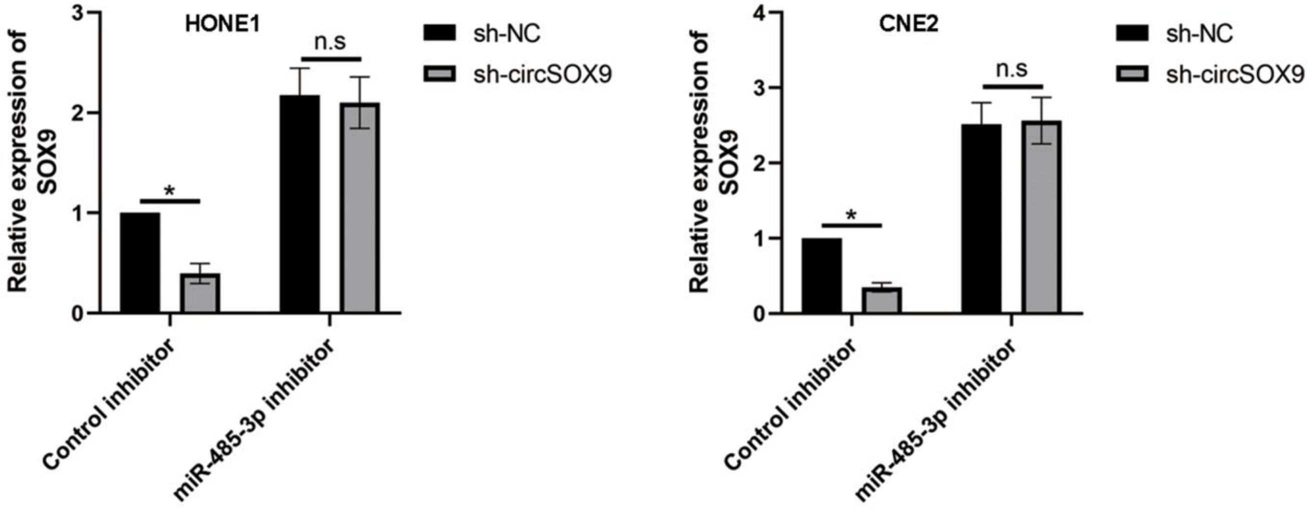

Supplementary Figure 2. qRT-PCR analysis of the SOX9 expression in cells with knockdown of miR-485-3p. $^{*}P < 0.05$.
